# Celebrity Politics and Democratic Elitism

**DOI:** 10.1007/s11245-021-09763-0

**Published:** 2021-10-11

**Authors:** Alfred Archer, Amanda Cawston

**Affiliations:** Department of Philosophy, Tilburg University, PO Box 90153, 5000 LE Tilburg, The Netherlands

**Keywords:** Celebrity, Epistemic power, Democratic elitism

## Abstract

Is there good reason to worry about celebrity involvement in democratic politics? The rise of celebrity politicians such as Donald Trump and Vladimir Zelensky has led political theorists and commentators to worry that the role of expertise in democratic politics has been undermined. According to one recent critique (Archer et al. 2020), celebrities possess a significant degree of epistemic power (the power to influence what people believe) that is unconnected to appropriate expertise. This presents a problem both for deliberative and epistemic theories of democratic legitimacy, which ignore this form of power, and for real existing democracies attempting to meet the standards of legitimacy set out by these theories. But do these critiques apply to democratic elitism? In this paper, we argue that recognition of celebrity epistemic power in fact represents a valuable resource for supporting the legitimacy and practice of democratic elitism, though these benefits do come with certain risks to which elite theories are particularly vulnerable.

## Introduction

The election of celebrities like Donald Trump (USA) and Vladimir Zelensky (Ukraine) to national presidencies has reignited discussions about the role of celebrities in democratic politics. These discussions are nothing new. In the 1950s, C. Wright Mills (1957: 62), criticized the role that celebrity played in American society, arguing that it was necessary to enter the world of celebrity in order to achieve power and prestige. The phenomenon of celebrities entering the political arena has also been criticized for turning politics into a sector of the entertainment industry (Postman 1987), for leading to appearance and presentation being prioritized over the ability to govern (Meyer 2002: 79) and tilting the political balance in favor of the rich and powerful (West and Orman 2002: 113). More recently, Archer et al. (2020) have argued that celebrity involvement in democratic politics is problematic because celebrities possess significant levels of epistemic power (roughly the power to influence what people believe) that is unconnected to any relevant expertise. This, they argue, creates a problem for deliberative and epistemic theories of democratic legitimacy, as these theories do not take this form of power into account. It is also a problem for any democracy attempting to live up to the ideals articulated by these theories, as this is a further challenge any society must overcome in order to meet these ideals.

Our aim in this paper is to investigate whether the epistemic power possessed by celebrities also creates similar problems for democratic elitism, the view that society should be ruled by political elites but that these elites should be subject to democratic contestation. An important issue for democratic elitism to address is how to manage the epistemic divide that may exist between the political elites and the people they are governing. We will argue that recognition of celebrity epistemic power in fact represents a valuable resource for supporting the legitimacy and practice of democratic elitism, though these benefits do come with certain risks to which elite theories are particularly vulnerable.

We will begin in Section One by outlining the phenomenon of celebrity epistemic power. We will then, in Section Two, outline democratic elitism. In Section Three, we will argue that the epistemic power of celebrities may play a valuable role in democratic life from the point of view of democratic elitism. Celebrities can serve as a check on the power of political elites, they can direct attention to those who tend to be ignored by such elites and they can help facilitate public engagement with complex political issues. However, in Section Four we will consider reasons for democratic elitists to be concerned about the role of celebrity epistemic power. The celebrity industry may prevent celebrities from being able to hold governments to account in a fruitful way, and there is a risk of continuous competition for attention, and the subversion of competitive (meritocratic) mechanisms for selecting elites.

While the academic literature on this topic tends to be critical of the impact of celebrities on democratic politics (Eg. Postman 1987; Meyer 2002; West and Orman 2002), there are those who offer a qualified defense of celebrity politicians. John Street (2004), for example, argues that the existence of celebrity politicians is compatible with a coherent and plausible account of political representation. Our aim is to offer a similarly qualified defense of the role of celebrities in democratic politics by examining how celebrities can serve to bridge the epistemic divide between the people and the political elite. Celebrities can play a role in preventing this divide from growing too large and can serve as useful communicators across this divide. However, celebrities may be prevented from playing these useful roles by the celebrity industry that underlies our contemporary star system. By making this case, this paper will make an important contribution to our understanding of the role of celebrities in democratic politics.

This paper will also make an important contribution to the literature on democratic elitism. In particular, this paper will develop existing discussions about democratic elitism’s relevance for actually existing democracies. In the introduction to their edited volume on democratic elitism, Best and Higley (2010) argue that democratic elitism overlooks important features of actual democracies such that its continued relevance and utility are called into question. In particular, they argue that Schumpeterian theories oversimplify the impact of elite control of competitions on the democratic values such theories intend to reconcile with elitism. They identify two recent trends on this point that democratic elitism must address to demonstrate modern relevance. The first trend concerns the growing public interest in leaders’ personal charisma and likeability, and that these factors (rather than competence or experience) increasingly influence competitive outcomes. As Best and Higley (2010: 12) write, “leaders who inspire trust, project strength, and gain wide appeal define issues, overshadow party platforms and dominate elections that are preponderantly referendums on competing leaders’ images”. This focus on the leader has displaced the role of the party in vetting political elites and in setting agendas: “Leaders now bring parties to power rather than the other way around” (Best and Higley 2010: 13). Best and Higley attribute this change, in part, to increased political uncertainty and growing complexity of political problems including globalization and climate change (2010: 14). Consequently, voters are drawn to leaders perceived to be individually flexible and capable of dealing with future problems, and who can subsume complex issues under easy slogans. This ‘dominance of political leaders’ (2010: 11) risks removing leaders from the regulative control of colleagues, bypasses the screening mechanisms of party politics, and departs from the consensually united elite restrained partisanship model of political elitism (2010: 7–8) that can work to protect democracy.

The second, and related, trend concerns the ‘symbiotic’ relation between mass media and political leaders (Best and Higley 2010: 14). They note the power of the media to portray and communicate leader personas, and the subsequent investment leaders make in image-management and PR consultants. Moreover, mass media’s commercial interests drive its preference for ‘brevity, drama, and simplicity’ over sustained debate, and thus further promote focus on charismatic leaders. According to Best and Higley (2010: 14) leaders thus perform as, or even become *celebrities*: “Conscious of the impact that personalized media exposure has on voters, leaders comport themselves as, and are at the same time accused of being *celebrities*” (our emphasis). Together, these two trends point to important challenges that contemporary democratic elitism is called on to address. In this paper, we further develop the worries expressed by Best and Higley concerning these trends, while also revealing ways in which these trends may provide resources for democratic elitists.

## Celebrity and Epistemic Power

In a recent paper, Archer et al. (2020) argue that celebrities possess epistemic power that is divorced from expertise and, as a result, celebrity involvement in politics threatens democratic legitimacy. In this section, we will explain this critique before going on to consider in the remainder of the paper the extent to which this critique creates problems for democratic elitism specifically.

How should we understand the term celebrity? According to a number of those involved in the academic study of celebrity, the central feature of celebrity is being known.

As Antoine Lilti (2017: 6) puts the point:

The celebrated individual is not known simply to his family, his colleagues, his neighbours, his peers or his customers, but to a vast group of people with whom he has no direct contact, who have never met him and will never meet him, but who frequently encounter his public image.

While people may become known for a particular talent, role or profession, celebrities are often known in ways that go beyond the reasons why they became famous in the first place. According to Daniel Boorstin’s (1962: 57) influential definition, a celebrity is “a person well-known for their well-knownness”. Importantly for our purposes, celebrities are not simply well known, they are also the focus of public attention. Robert Van Krieken defines celebrity as “a quality or status characterized by a capacity to attract attention, generating some ‘surplus value’ or benefit derived from the fact of being well known (highly visible) in itself in at least one public arena” (2012, 10). Celebrities then are those to whom large amounts of attention are paid in ways that go beyond their specific talents, expertise or professional role (Archer et al. 2020: 28).

It is worth noting that when we are discussing celebrity involvement in politics there are several different phenomena we may have in mind. ‘t Hart and Tindall (2009: 258) provide a useful categorization of the various forms of celebrity involvement in politics. First there are *celebrity advocates*: celebrities who advocate for a particular political issue, such as the U2 singer Bono’s anti-poverty campaigning. Second, there are *celebrity endorsers*: celebrities who provide support for political parties or candidates who are running for office, such as Oprah Winfrey who publicly supported Barack Obama’s bid for the US presidency in 2008. Third, there are *celebrity politicians*: celebrities who run for political office. For example, Donald Trump who became famous as a billionaire and TV-personality before becoming a politician. ‘t Hart and Tindall’s final category are *politician-celebrities*: politicians who become celebrities through their work as a politician, either by accident or design. Barack Obama, for example, might be seen as someone who became a celebrity through his role as a politician. In addition to ‘t Hart and Tindall’s taxonomy, we propose including *activist-celebrities:* these are political activists who become celebrities through their activism, for example, environmental activist Greta Thunberg. Finally, celebrities may influence politics in other indirect ways, for instance by publicly commenting on certain issues or promoting certain beliefs. For example, comedian Bill Maher is not an anti-vaccine activist, but his 2009 comments opposing mandatory vaccination, citing distrust of the government (Whitelaw 2009), may have influenced public opinion on vaccination and contributed to the politicization of the issue.

The next concept to explain is that of epistemic power. This, roughly, is the power one has as an epistemic agent to influence other epistemic agents. To put this in less technical terms, it is the power one has as someone with opinions, beliefs and knowledge to influence what other people think. The definition given by Archer et al (2020: 29), which is based on the earlier definition of Geuskens (2018), is as follows:

*Epistemic Power:* A person has epistemic power to the extent she is able to influence what people think, believe, and know, and to the extent she is able to enable and disable others from exerting epistemic influence.

For example, a journalist possesses epistemic power, as she will be able to influence the beliefs of her readers. She will also be able to direct her readers’ attention towards other people and in doing so enable them to exert epistemic influence.

Celebrities possess at least two sources of epistemic power (Archer et al. 2020). First, many celebrities will be perceived as more credible than other people, at least among certain groups. Credibility is an important source of epistemic power, as the more likely people are to believe what one says, the more influence that person has to influence the other’s beliefs. The idea that celebrities are seen as more credible than others underlies the use of celebrities in advertising and political campaigns. However, the evidence about the effectiveness of this use of celebrity is rather mixed. Celebrity endorsements have been found to have a positive influence on consumer attitudes towards certain products but no significant general effect on consumers’ intentions to actually buy the products. The effectiveness of celebrity endorsements appears to depend upon their gender, profession and their perceived fit with the product (Knoll and Matthes 2017). The evidence of the effectiveness of celebrities in political campaigning is also mixed. Again, the effectiveness of the endorsement appears to depend on a complex link between the celebrity, the opinion they are endorsing and the audience they are speaking to (Jackson and Darrow 2005: 94). Moreover, celebrity political endorsements have been found to be especially effective amongst those who spend little time thinking about politics (Veer et al. 2010) and amongst young people particularly on social issues rather than economic ones (Becker 2010: 112–116). Finally, celebrity endorsements have been found to be particularly effective on fans of the celebrity (Jackson and Darrow 2005). There is then good reason to think that celebrities are viewed as more credible than others, at least amongst certain groups of people.

The second source of epistemic power is attention. As we have already pointed out, a distinguishing feature of celebrity is that celebrities are paid attention to in a way that other people are not. In the words of David Marshall, celebrities function as “a voice above others” (1997: xlviii). Moreover, they are paid attention to in ways that go beyond their particular expertise, role or profession. Being a focus of attention is an important source of epistemic power for celebrities that is distinct from credibility. Those who have the attention of others have a platform for their testimony to be heard. Without this platform, it does not matter how credible people would view their testimony, as it will not be heard. Being the focus of attention also gives celebrities the ability to redirect attention to others. This means that even if people do not find the celebrity credible the celebrity may still be able to influence their beliefs by directing their attention towards people they do find credible. Being the focus of attention also gives celebrities significant power to influence political agendas. For example, a large-scale media analysis of the 2016 U.S. election found that Donald Trump used his celebrity profile to effectively set the political agenda. While media coverage may not have reflected Trump’s views, it focused on the issues Trump wanted to focus on (Faris et al. 2017: 5).

Celebrities, then, often possess significant amounts of epistemic power due to being seen as credible, at least among certain groups of people, and for being the focus of attention. This gives all celebrities special power to influence the democratic process, whether they do so as an activist, an endorser or as a politician. Politician-celebrities and activist-celebrities also benefit from these special powers of credibility and attention.^1^ Moreover, this power is not connected to any special expertise or professional role. As Archer et al. (2020: 31) point out, this power creates a challenge for democracies seeking to live up to the standards articulated by two of the central theories of democratic legitimacy. According to *deliberative* theories of democracy, the legitimacy of democratic rule is grounded in public debate between citizens who respect each other as moral equals and who do not allow asymmetries of power or resources to unfairly influence the deliberation (Habermas 1975; Knight and Johnson 1997). *Epistemic* theories of democracy add to this that part of the legitimacy of democratic rule comes from the epistemic value of these procedures (Estlund 2007; Peter 2007; Young 2000). Archer et al. (2020: 33–35) present three problems that the epistemic power of celebrity presents for democracies seeking to live up to either of these democratic ideals. First, celebrities can use their epistemic power to center public discussion on the issues that they find important and to frame public debates in ways that suit them. This agenda setting power gives them an unfair degree of influence in public deliberation. Second, celebrity epistemic power is not subject to the usual checks on power designed to prevent other forms of power leading to undue influence in democratic deliberation. Third, the fact that celebrity epistemic power is not linked to any special expertise or professional role threatens the epistemic value of democracy by undermining the ability of democratic deliberation to track the truth and to produce knowledge.

Our aim in this paper is not to evaluate the problems that celebrity epistemic power raises for deliberative and epistemic theories of democracy. Instead, our aim is to consider whether this power also creates problems for theories of democratic elitism.

## Democratic Elitism

Democratic elitism aims to find a middle ground between classical elitists, such as Vilfredo Pareto (1935), Gaetano Mosca (1939), and Robert Michels (1966) and more idealist forms of democracy such as those defended by Alexis de Tocqueville (1840) and John Stuart Mill (1859). Classical elitists held that democracy is unworkable in modern society and so society should be ruled by elites. In contrast, democracy holds that society should be ruled by the people in an equal and inclusive way. Drawing from both these views, democratic elitism holds that society should be ruled by political elites but that these elites should be subject to democratic contestation.

The appeal of democratic elitism lies in the idea that granting full power to the people may lead to an illiberal and unjust society. Joseph Schumpeter (1942: 242) asks us to consider a country that, through democratic means, subjects a minority of its citizens to persecution. Should a democrat approve of this country? Schumpeter claims that they should not and what this shows is that there are ideals and interests, such as the protection of liberty, that democrats should hold to be more important than the democratic process. In order to ensure that these ideals and interests are secured, a society needs to find leaders who are capable of pursuing these ideals. The way to do so is to ensure that political leaders are chosen from an elite social group who are able to incorporate high performing individuals from the lower classes. This is an elitist view then, as countries will be run by political elites. However, it is also democratic, as citizens are able to choose their leaders from these elites. Rather than “government by the people”, Schumpeter claims we should aspire to “government approved by the people” (Schumpeter 1942: 269). This can still be seen as a form of democracy, according to Schumpeter, as “Democracy means only that people have the opportunity of accepting or refusing the men who rule over them.” (Schumpeter 1942: 285).

Schumpeter goes on to specify four conditions required for a government on this model to be successful. First, the pool of candidates engaged in professional politics must be of ‘sufficiently high quality’, which, as mentioned above, is likely achieved by the existence of social class structures (1942: 257). Second, democratic methods are appropriate only for indicating general values and ought not replace specialist or expert knowledge. Legislation on complex issues should be protected from irrational influence from either the public or parliament (1942: 257). Third, and relatedly, the government must be supported by a competent bureaucracy (1942: 260). And fourth, citizens must exercise ‘democratic self-control’ meaning they must not interfere with governing once they have selected their representatives. Government must be autonomous and free from “political back-seat driving” (1942: 262).

In an influential critique of democratic elitism, Peter Bachrach (1967: 105–6) raises a number of problems with the democratic elitist solution to the problems facing the illiberal and anti-democratic potential of democracy. First, there is no reason to assume that elites will be any more inclined to protect liberal democracy than other citizens, especially if doing so may put their elite status at risk. Second, trying to combine elitism and democracy may destroy “the boldness and imaginativeness characteristic of democracies of the past.” (Bachrach 1967: 106) Third, the competing interests among the elite means that they will find it difficult to establish a sufficient consensus to allow them to safeguard democracy when it is under threat. Relatedly, even when elites manage to reach such a consensus among themselves, they will then face the further challenge of having to create democratic support for this consensus.

The underlying problem here, is summarized by Bachrach in the following:

If it is time to abandon the myth of the common man’s allegiance to democracy, it is also time that elites in general and political scientists in particular recognize that without the common man’s active support, liberty cannot be preserved over the long run. The battle for freedom will be lost by default if elites insulate themselves from the people and rely on countervailing forces, institutional and social barriers, and their own colleagues to defend the system from the demagogic leader of the mob. Democracy can best be assured of survival by enlisting the people’s support in a continual effort to make democracy meaningful in the lives of all men. (1967: 106).

In other words, the challenge for a democratic elitist form of government would be to ensure the active support and participation of citizens in democratic life. If elites attempt to isolate themselves from other citizens then democracy is unlikely to mean anything to ordinary citizens and, as a result, is unlikely to survive.

In recent work, Alfred Moore (2017) has defended a view that he calls “critical elitism’, which seeks to provide a form of democratic elitism that can adequately respond to this challenge. According to Moore, one of the major challenges to democratic ideals is the need for citizens to rely upon experts both as a source of testimony and of practical knowledge. In the COVID-19 pandemic, for example, public health experts have to be relied upon for their ability to make good judgements about the safety of vaccinations and also for their practical knowledge of how to run an effective vaccination program. This reliance on expert authority, however, seems to be in tension with the democratic ideals of equality and the public questioning of authority. As Moore (2017: 2) describes the tension: “We clearly need scientific and expert authority in order to formulate considered collective judgements and carry out collective decisions. Yet public questioning, criticism and rejection seem to make such authority even harder to sustain.”

The aim of critical elitism is to find a way in which expert authority can be made compatible with democratic ideals. The view consists of three core claims. First, expertise always involves inequality, as it involves one group of people (non-experts) recognizing that another group (experts) possess knowledge, skills, information and expertise that they (the non-experts) do not (Moore 2017: 6). Second, citizens need not always actively participate in expert decision-making (Moore 2017: 7–8). An active decision not to participate in certain areas and to defer instead to experts also has an important role to play in democratic society. Third, Moore (2017: 8) claims that expertise must be authoritative and that this authority arises through public challenge and contestation. While it is legitimate to defer to expert authority on certain issues, this expert authority must be continually subjected to the critical judgement of the citizens. Overall then, the key thought behind critical elitism is that expert authority is compatible with democratic ideals when it is subject to continual public contestation.

An alternative response to Bachrach’s call for citizen participation can be found in Jeffrey Green’s (2010) theory of plebiscitary democracy. Green agrees that the familiar understanding of democracy as a system based on the people’s voice fails to track the reality. Rather, citizens in today’s democracies are better described as spectators rather than participants. However, Green argues that this spectatorship can be compatible with democratic ideals. This compatibility is based on redefining the subject and source of citizen power. The subject, argues Green, ought to shift from a focus on legislation, to leaders, in particular, to a focus on leaders’ character and conduct. The source of power shifts from participation and exercise of voice, to observation and surveillance akin to a disciplinary ‘gaze’ (2010:9). Citizens do not communicate their views on policy to politicians who are motivated to listen, rather citizens observe and asses the performance of political leadership. For this relation to remain democratic, Green argues certain institutional checks are necessary. These checks again do not focus on political decision-making, but on how the performance is presented. Plebiscitary democracy requires citizens have significant “control of the means of publicity” (2010:14) so that leaders cannot wholly manipulate the object of citizens’ gaze.

## A Useful Role for Celebrities?

In section one, we explained a general critique that can be raised about celebrity involvement in democratic politics; celebrities possess high levels of epistemic power that is unconnected to any form of expertise. This is a problem for theories of democracy which ignore this form of power and for any existing democracy aspiring to live up to democratic ideals. We have explained this critique in relation to deliberative and epistemic theories of democratic legitimacy. But does the critique also create problems for democratic elitism? Having outlined the theory of democratic elitism, we are now in a position to assess whether celebrity epistemic power is also problematic according to this theory of democratic legitimacy. We will argue that celebrity epistemic power in fact represents a valuable resource for supporting the legitimacy and practice of democratic elitism. We will do so by outlining three worries that might be raised about societies run along democratic elitist lines and, for each worry, explaining how the epistemic power of celebrities could help to alleviate these concerns. Our interest here concerns the challenges that would be faced by a society that attempted to live up to the ideals of democratic elitism, rather that challenges facing existing societies.

The general concern underlying all three of the worries we will outline here is that a society run in a democratic elitist way would create a professional political class with a distinct outlook on the world and a distinct set of interests and concerns. This is the core idea of democratic elitism, that political leaders should be chosen democratically from within an elite social group and that this will help to ensure the protection of justice and liberty.

The first reason to worry about the creation of such a class is that this group will possess too much power that is not subject to external scrutiny. Of course, a democracy functioning along democratic elitist lines would involve the public contestation of ideas from within this political class. Moreover, such a society would allow the people to choose whom they want as their leader from within this class. This public contestation of ideas and public selection of leaders is needed to qualify a political system as a form of democratic elitism rather than non-democratic elitism. However, on this model the ideas that are publicly contested and the people competing for political leadership would all arise from within this political class. This means that while the individuals and groups within this class will be subject to checks on their political power, the political elite as a whole will not. In such a situation, there is reason to worry that the political elite will promote their own group interests rather than the interests of society as a whole, as the only ones providing a check on this power will be other members of this elite.

This, of course, is a simplified picture. In reality, the political elite must answer to a number of other groups of elites. The media, business leaders, academics and other forms of expert can all play a role in holding the political elite to account and in publicly contesting the ideas they put forward. However, it remains the case that a group of professional elites will be held to account, for the most part at least, only by professional elites. The role of citizens outside of these elites lies simply in deciding which of these members of these elites become political leaders. The worry remains then that these elites will be motivated primarily in protecting elite interests rather than in protecting the interests of citizens more generally.

The first reason to think that celebrity epistemic power may have a useful role to play in democratic life from the point of view of democratic elitists is that it can serve as an important check on the power of a professional political class. As Francesco Alberoni (1962: 75) observes, there are two kinds of people in society who “are especially remarkable and who attract universal attention.” The first group are those with institutional power who attract attention because their decisions have a major impact on society. This includes, amongst others, political leaders, the CEOs of major companies, high profile government advisors and the owners of major media organizations. The second group are celebrities, including musicians, actors and sporting champions. This group lack institutional power and their decisions do not have a major impact on society. Nevertheless, stars are paid attention to because people admire them (Alberoni 1962: 90). Alberoni’s focus is on examining the useful role that celebrities can play in society in meeting the needs for community identification in large-scale societies. He argues that it is useful for those living in large societies to have people who function as shared objects of gossip and that celebrities meet this need. However, the existence of an elite group separate from the elite that wield institutional power may also serve a different function. The epistemic power that celebrities possess by being objects of admiration and attention means they are well placed to serve as an external check on the power of the group of elites who possess institutional power. Celebrities, then, could play an important role in democratic elitist societies by providing an external check on the power of the political elite.

It is worth noting that this point will only apply to certain kinds of celebrity involvement in politics, namely celebrities who are famous for non-political reasons who seek to make an impact on politics. Those who have become celebrities through their political work, such as Boris Johnson or Barack Obama, cannot serve this function, as they are too embedded within political elites. This role then is only available to celebrities who are not integrated in the circles of the elites with institutional power. Moreover, they can only play this role for as long as they remain outside of this group. This is an important point, as celebrities who become politically engaged often become integrated within the political elites. This is clearly the case with celebrities who become politicians and so must engage in party politics. Even celebrity activists, though, may end up too integrated in political elites. For example, Heribert Dietmar and Rajiv Kumar argue that U2 singer Bono’s anti-poverty activism became essentially a public vehicle for the views of Jeffrey Sachs, economics professor and president of the United Nations Sustainable Development Solutions Network. As Dietmar and Kumar (2008: 261) describe this relationship, “Bono and Sachs have become something of a double act, with the professor providing the intellectual message and the rock star bringing it to large audiences.” While becoming integrated with political elites may be useful for advancing certain political ends, it undermines the ability of celebrities to serve as an external check on the power of political elites. Nevertheless, the epistemic power possessed by celebrities allows them to play this role so long as they can remain largely separated from those with institutional power. In doing so they can play a useful role from the point of view of democratic elitism by ensuring that the political class are subject to external checks on the use of their power.

The second reason to worry about society being run by political elites is that there is a risk that this group will be cut-off from the concerns of ordinary people. If politicians are drawn entirely from an elite group of political professionals then there seems good reason to worry that they will not share the same concerns as other people and will not be in a good position to understand what matters to them. This means that even if they are sincerely trying to make decisions for the good of society as a whole, they may make the wrong decisions due to a lack of awareness of what people outside of the political elite care about. This is an important problem from the point of view of democratic elitism, as the political elites are supposed to be those who are best able to govern in the interests of all. If they are unable to understand the interests of those outside of these elites, then they are unlikely to be capable of governing in the interests of all. As Moore discusses this problem in relation to his critical elitism, non-elites may have relevant local or practical expertise that is overlooked or marginalized by those with expertise (2017: 88–89).

Celebrity epistemic power can also play a useful role in helping avoid this problem by directing the attention of political elites and experts towards the cares and concerns of those who tend to be ignored by the political class. Because they exist outside of this political class, they may have contact with marginalized people that members of the political elite do not. Celebrities can then use their epistemic power to help give voice to their concerns. For example, English footballer Marcus Rashford was able to use his celebrity status to campaign successfully for the UK government to extend access to free school meals for low-income children during the COVID-19 pandemic. As a 22 year-old who had grown up in poverty, Rashford had direct access to the concerns of those facing poverty in a way that many members of the political class did not. Rashford’s credibility, combined with his platform as a famous footballer, enabled him to generate significant interest in the issue of food poverty, and over a million people signed his petition on the issue within 2 weeks of its launch. In this way, he was able to direct the attention of both politicians and the public towards the struggles of people the political class were ignoring. The ability of celebrities to play this role is important from the point of view of democratic elitism, as it can help to counteract the problem of political elites being out of touch with the concerns outside of their group.^2^

The third reason to worry about a society run by political elites with a distinct outlook on the world is that this may make it difficult for the political class to communicate their ideas to the wider public. The professional political class may be so used to dealing with complicated political issues that they may struggle to translate these ideas into a message that the public can understand. Relatedly, the political elites may simply appear unrelatable to those outside of this class. This is a problem for a society aspiring to democratic elitist ideals, as it is important that citizens make informed choices when engaging in the (limited) democratic decisions that they are participating in. As Moore (2017: Ch.5) points out, for citizens to make judgements on expert decisions or debates, it is important that these issues can be articulated in a way that they can understand. If citizens are unable to make sense of the decisions they must make or are unable to relate to any of the available political candidates, then they are unlikely to engage in the democratic process in an informed way.

One way to respond to this problem is for politicians to invest a lot of time and energy in developing messages the public can understand and relatable public personas. While there is some value in this, time invested in making the political class understandable and relatable is likely to take away from the time politicians can spend on the task of governing their country. This is also important from the point of view of democratic elitism, as one of Schumpeter’s (1942: 262) conditions for an effective form of democratic elitism is that there be a division of labor between the voters and the politicians. This means, according to Schumpeter, that voters should allow politicians sufficient autonomy to do their jobs effectively and should not bombard them with so many instructions to the extent that they are engaging in “political back-seat driving” (1942: 262). It also means, though, that politicians should not have to devote too much of their time to explaining the decisions they are making and developing a relatable public persona, as this too distracts from the business of governing.

Again, the epistemic power of celebrities has a potentially useful role to play in helping societies overcome this challenge and live up to the ideals of democratic elitism. As John Street (2004) has argued, politicians in today’s societies need to draw on resources from popular culture to help represent the political to their audiences. As Street (2004: 446) puts the point, “Political representation is an art that draws on the skills and resources which define mass-mediated popular culture.” To varying extents then, according to Street (2004: 446), all politicians make use of the techniques from popular culture in order to “condense ‘the political’” for those they represent. In simpler terms, politicians need to be able to create a public persona that can represent their policies in an effective way (Corner 2000: 401). If a politician is standing for a more compassionate form of politics, it is important that their public persona is one of a compassionate person who may allow themselves to display vulnerability in public. On the other hand, a politician aiming to bring in strict law and order policies should develop a strong public persona and guard against any perceptions that they are weak or vulnerable. In this way, the politician can make the political choice tangible to the audience. This skill of developing a public persona that represents certain beliefs, values and emotions is one of the key skills possessed by celebrities. As David Marshall (1997: 203) puts the point, “in politics, a leader must somehow embody the sentiments of the party, the people, and the state. In entertainment, a celebrity must somehow embody the sentiments of the audience.” This skill can be seen as a form of epistemic power, as it is an ability to present complicated issues to people in a way they can understand and so influence their understanding of the situations.

Given this talent that celebrities possess, they have a potentially helpful role to play in helping societies live up to the ideals of democratic elitism. Celebrities can use their expertise in this to help facilitate public understanding of politics. Celebrities will also be well placed to serve as representatives of political values and character and, following Green (2010), to focus citizen attention and scrutiny on these ideals rather than the details of policy. Building on Schumpeter’s idea of a division of labor between voters and politicians, we could imagine a division of labor between politicians who focus on the business of governing and celebrity politicians who focus on the business of representing politics and political values to the public. This would enable those doing the governing to focus on this task, whilst the celebrity politicians enable the public to make sense of the political choices they face. This is true both for career politicians who become celebrities and for celebrities who become career politicians. In both cases, the skills these celebrities possess can be used to allow for a public contestation of political ideals that the public are able to engage with.

In summary, the epistemic power of celebrities represents a valuable resource for a political system attempting to live up to the ideals of democratic elitism. This power can enable celebrities to serve as an important external check on the power of the political elite, it can be used to direct attention towards those who are likely to be ignored by such an elite and they can help facilitate public engagement with complex political ideas. These considerations give us reason to think that democratic elitists may have less reason to worry about the role of celebrities in politics than other theories of democracy.

## Celebrity Politics as a Problem for Democratic Elitism

However, there are several reasons for democratic elitists to worry about celebrity involvement in politics. In this section, we discuss two such concerns: (1) the limits on celebrity abilities to counter elite interests and (2) the risks of continuous competition for attention, and the subversion of competitive (meritocratic) mechanisms for selecting elites.

As mentioned above, celebrities outside of the political system could function as checks on power and as conduits through which public interests are brought to the attention of elites. These mechanisms share similarities with some understandings of the mass media as a ‘go-between’ that mediates interactions between the public and politicians, and as an independent ‘watchdog’ holding government to account. However, these mediator and watchdog roles for the media have been criticized, and some of these criticisms apply to the case of celebrity. Gulbrandsen (2010), for instance, in his review of the relation between politics and mass media in Norway, observes that politicians report positively on the media’s ability to communicate issues to the public but are also wary of the media’s ability to interfere with governing (limiting politicians’ autonomy). They try to ‘defend themselves’ from the media by strictly managing interactions, for example by implementing formal channels for inquiries or dissemination of information and by incorporating communications professionals and ‘spin’ tactics. Ultimately, Gulbrandsen claims the media’s watchdog function is increasingly at risk, leading to ‘a democratic elitism that is increasingly elitist in its working’ (2010:127).

There is a parallel worry to explore in the case of celebrity influence and the ultimate control the political sphere has over media influence. It is important to question where ultimate control lies in the case of celebrity influence and how this affects its potential watchdog effect. While many celebrities today enjoy more freedom in how to set and manage their image than celebrities of the past, it is important to acknowledge the ongoing industry of celebrity and the commodification of ‘personalities’.Achieving celebrity status requires significant investment, and the resulting commodity is highly valued. This is true whether the personality is cultivated and owned by an organization (e.g. record label or production studio), or pursued by individual agents who hire their own image consultants and management. Consequently, it is important to recognize that celebrity, as a contemporary, capitalist phenomenon, may not represent a progressive agenda. As David Marshall (1997: xlviii) argues, “the celebrity as public individual who participates openly as a marketable commodity serves as a powerful type of legitimation of the political and economic model of exchange and value—the basis of capitalism.” Rather, it is likely that the interests of the political elite broadly align with those of the corporate elite. If so, there is a risk that celebrity appears as an outside, competitive check, when in practice, there is collusion. This worry is reminiscent of Schumpeter’s critique of the idea of the ‘will of the people’. Given the success that advertisers have in influencing what people want, there is little sense in relying on the idea of the ‘people’s will’. The ‘people’s will’ more plausibly reflects a manufactured will, “…the will of the people is the product and not the motive power of the political process” (1942: 236). While Schumpeter’s characterization of the people’s will as wholly manufactured may be too strong, the weaker claim that the people’s will is largely manufactured seems reasonable and can be extended to the subject of celebrity. Thus, public interest in celebrities could be understood as largely produced rather than the primary originating force. Celebrity is not the product of the people’s attention, rather, it reflects the result of largely manufactured attention. If so, it is not clear whether celebrities reflect the interests and concerns of their public, or, as commodities, whether the epistemic power they command promotes the interests of certain groups.

The second worry concerns the role of competition for power in democratic elitism. One way to spell out Best & Higley’s worry about increasing focus on leaders, and their celebritization, is the risk that celebrity leaders may be perceived as competent on matters that they are not as a result of credibility-creep. Donald Trump’s celebrity, for instance, was based initially on his status as a successful businessman and his role on a related reality TV show. But despite having no political experience, many believed Trump would be sufficiently competent to handle complex international relations, run a national economy, and protect citizens during a global pandemic. Trump may be an odd case, as not many are elected to top positions of political power with zero previous experience, but the case illustrates the potentially wide extent to which credibility creep can operate, and the high stakes involved. Trump’s campaign also illustrated the power of leader politics to bypass or circumvent the usual party-level checks that help ensure a competent outcome. Schumpeter’s faith that there are “many rocks in the stream that carries politicians to national office which are not entirely ineffective in barring the progress of the moron or the windbag” (1942: 256) may need to be revisited.

But there is a second issue here as well. Best and Higley’s (2010) concern about leader-dominated politics that trade on charisma and sensationalization (aided by the media) bordering on celebrity suggests the nature of the competition has changed. Competition in democratic elitism was previously conceived as competition for public approval which ultimately translated into votes and political power. However, since a key element of celebrity epistemic power, i.e. the power to influence what others think, is tied to attention, it is plausible that competition for attention will become an important aspect of political competition for approval. This has three potential negative consequences for democratic elitism. First, a shift to attention rather than approval further disconnects the relevant competition from producing competent elites. From the point of view of Moore’s critical elitism, it would lead to public debates and decision making that are not shaped by the relevant expertise. And second, it promotes continuous competition. Schumpeter warns against continuous competition as it incentivizes politicians to value policies with short term benefits over long term, i.e. to make poor governance decisions. But a trend towards increased celebritization of leaders will put a related pressure on politicians to engage in constant competition. This is because a celebrity is one who is well known. And, measured, sensible policy is unlikely to attract as much attention as provocative, emotionally charged, or polarizing policy. This points to the third worry, namely that continuous competition for attention, disconnected from competence and party checks, and incentivized to provoke, is highly unlikely to result in governance that protects democratic ideals.

These concerns about the changed nature of competition and the focus on attention also raises problems for Green’s plebiscitary democracy. First, Green (2010) argues that political leaders should be held to account through ‘candid’, unscripted public events. Given that celebrities are particularly adept at presenting themselves in ways that appear authentic (Click et al. 2013) there is reason to worry that this mechanism of accountability will hand a distinct advantage to celebrity politicians over their competition (Archer et al. 2020). Second, Green’s call for institutional checks for authenticity and unscripted moments suggests he believes citizens critical gaze is focused on evaluating character and personality. However, the above discussion indicates that celebrity leaders will have a primary interest in attracting attention, which can then be used to cultivate approval. There is no guarantee that the citizen gaze will focus on character over bombast and spectacle and thus no guarantee that the outcome of the competition for citizen attention will translate into competent or virtuous leadership. As suggested above, there may even be incentives to depart from the familiar checks on competence for its shock value and subsequent draw of attention.

## Conclusion

A key challenge facing democratic elitism is how to manage the epistemic divide that may exist between the political elites and the people they are governing. We have argued that celebrity involvement in politics can play a useful check on the power of political elites, which can help to prevent this epistemic divide from becoming too wide. In addition, celebrities can serve as useful communicators across this divide. Celebrities can alert elites to the concerns of the people and can present the political debates and divisions taking place within the elite to the public in an engaging and accessible way. However, the celebrity industry and the interests of those involved in this industry may prevent celebrities from playing this useful role. Moreover, increased celebritization of politics may incentivize harmful and ongoing forms of competition that undermine norms of good governance that legitimize democratic elitism.

This conclusion has important implications for how we think of celebrity involvement in politics. While many have criticized celebrity political involvement for trivializing or distorting political life, we have argued that celebrities can play a useful bridging role between political elites and the people they represent. However, in order to play this role effectively they need to have a sufficient degree of autonomy and independence from those who run the celebrity industry.

Our conclusion also has important implications for the theory of democratic elitism. As Best and Higley (2010) note, contemporary theorists of democratic elitism need to consider how the theory ought to understand and respond to the twinned trends of leader-dominance and mass media influence. In our view, these trends are helpfully investigated through the lens of celebrity scholarship, and in particular, via the concept of celebrity epistemic power. A focus on celebrity epistemic power helps to underpin the worries initially expressed by Best and Higley and illuminate the nature of the problem. However, this analysis also reveals potential positive sites of influence. Celebrity influence may support the reconciliation of democracy and elitism by strengthening checks on elitist power, drawing attention to issues that would otherwise be ignored, and facilitating public engagement while protecting elite autonomy. We also pointed to distinct risks that democratic elitism faces from celebrity involvement, including how the nature of the celebrity industry may prevent celebrities from being able to hold governments to account in a fruitful way, and the risk of continuous competition for attention, and the subversion of competitive (meritocratic) mechanisms for selecting elites. In sum, while Best and Higley (2010) rightly direct our attention to the new threats that leader-dominance and media influence (celebritization) pose for democratic elitism, the confluence of these trends may also offer opportunities for revised forms of the theory.

## Footnotes

1 Though as Marsh et al. (2010) note, the different precise nature of this power may vary for the different categories of celebrity involvement in politics.

2 There are a number of important concerns that can be raised against the practice of celebrities speaking on behalf of marginalized groups, particularly if the celebrity is not a member of the group they are speaking for. See Smith and Archer (2020: 792) and Wallace (1990: 251) for discussions of the general problem of speaking for others. As this is not a distinctive problem for democratic elitism we will not investigate this issue here.
